# From health trackers to compasses: can wearables and apps deliver on their health prevention promise?

**DOI:** 10.3389/fdgth.2026.1967226

**Published:** 2026-10-09

**Authors:** S. M. Goodday, A. Goldenberg, F. Doshi-Velez, S. H. Friend

**Affiliations:** 1 4YouandMe, Great Neck, NY, United States; 2 Department of Psychiatry, University of Oxford, Oxford, United Kingdom; 3 Computer Science Department, University of Toronto, Toronto, ON, Canada; 4 Vector Institute, Toronto, ON, Canada; 5 Hospital for Sick Children, Toronto, ON, Canada; 6 CIFAR AI Chair, Vector Institute, Toronto, ON, Canada; 7 School for Engineering and Applied Science, Harvard University, Cambridge, United States

**Keywords:** apps, digital health (DH), digital health technologies (DHTs), disease, early intervention, health prevention, wearable and mobile computing

## Abstract

Despite long-standing knowledge of the significant contribution of lifestyle factors in the development of chronic conditions, lifestyle preventive strategies have failed to lower chronic disease rates globally [1,2]. Personal digital health technologies (P-DHTs) that can be worn on the person (wearables, smart devices and mobile phones) can be used for self-monitoring directly by the individual increasing health agency and access, with promise for transformation in health prevention. While these tools offer granular, continuous data on behaviours, emotions, physiology and stress, their passive information often fails to translate into actionable insights due to noisy signals and lack of individual level context. The P-DHT field has scaled P-DHT tools enormously over the past decade, but without scaling understanding. This perspective highlights how P-DHTs are currently shaping the health and wellness field as health trackers, and frames the opportunities and challenges in shifting these tools to the prevention field as ‘compasses’ where they are capable of making tangible changes in health outcomes.

## Introduction

### The problem

Chronic disease rates in North America have increased significantly—largely attributed to non-communicable diseases driven by modifiable lifestyle factors and a concerning upward trend in younger adults (1, 2). The major culprits behind this continued rise in chronic conditions are well known, cited, and discussed: 1) despite precision medicine initiatives, most healthcare approaches still use a ‘one-size-fits-all’, snapshot approach to researching, tracking and developing interventions for chronic conditions as opposed to a tailored, individualized approach that accommodates the dynamic nature of health; 2) chronic diseases are complex and multifactorial; 3) there are systemic barriers in access to and delivery of healthcare; and 4) there is a continued focus on acute care and management as opposed to prevention that targets modifiable lifestyle factors.

The precision medicine movement has touted to solve some of the aforementioned challenges, yet is optimized for a pharma/healthcare provider centric enterprise as opposed to a focus on patient empowerment. Huge advancements have been made in molecular profiling of individuals, but without understanding the end-user as a human person, living in the real-world with fluctuating capacities, constraints, and goals, personalization and precision of treatments lack meaning and understanding for patients and ultimately success.

With advancements in P-DHTs, new insights about personal day-to-day lifestyle, behavioral and psychosocial factors make a strong case for the integral importance of these domains to health prevention and management (3–5). We now have new objective measurement lenses that have made lifestyle and behavior visible in continuous, quantitative detail. Individualized patterns in physiology and other biometrics, sleep, physical and social activity, and our digital footprints showcase how important it is to understand health within an individual context, not through population averages (6–8).

The identification and focus of personal lifestyle factors as key to preserving health was adopted early in the 19th century (9), even before the field of risk factor epidemiology bloomed. While it has been widely reported for centuries that targeting lifestyle factors is instrumental in improving and sustaining health, why have we made such little progress in reducing chronic disease outcomes? There are certainly macro-level, socio-political factors at play, including the medical industrial complex that drives the interconnected network of private, profit-driven corporations that dictate health policy—pharmacological therapies that treat recurring symptoms (e.g., blood pressure lowering medications) make much more money than asking a patient to go for a walk (10). Moreover, individuals from disadvantaged backgrounds face real physical and social barriers in being able to engage in certain lifestyle changes (11, 12). However, these macro-level factors cannot account for this lack of progress in its entirety—what is the missing link? Is targeting lifestyle factors sufficient to instigate a health-related change, or are individuals just not motivated enough?.

Targeting lifestyle factors seems intuitively straight forward—be more active, sleep more, reduce stress, stay social, eat well. Relating back to the known ‘one-size-fits-all’ challenge—do we actually have the right tools to empower and engage an individual to truly understand ‘which’ lifestyle factors will make a significant outcome impact for them? For example, do individuals actually know when their stress is getting risky or what level of activity is beneficial for them? Targeting personalized lifestyle factors requires a level of understanding of what makes a unique individual better or worse over time, in relation to their dynamic external environment and their inherent strengths and vulnerabilities. Further, there is an assumption that individuals should be motivated to actually make a change. Yet, human behavior is deeply rooted by a need for immediate, short-term cause and effect feedback—a ‘present bias’ that overvalues short-term gratification and undervalues future results (13, 14). There is an adjacent existential challenge relating to the doctor—patient role and responsibility (4). Currently, patients are told (sometimes) by their healthcare providers to engage in health promoting behaviours during the constrained, infrequent clinical visit. It's not a surprise that the primary care physician will also struggle in engaging their patient to change their habits. Unless an individual is properly equipped with the motivational tools and has made a personal decision to change, simply being told to change a behavior is unlikely sufficient to promote long-term health related behavior change.

### The opportunity

P-DHTs are a powerful collection of modern tools with big yet immature ambitions. Most existing applications of P-DHTs have been in the fitness realm, tracking steps, and activities aiming to motivate a user to stay active, but in more recent years, many companies have pushed this boundary into the wellness technology field that extends to a more holistic approach to continuous monitoring of sleep, stress, and mental health (Oura, Whoop, Apple, Google). With a few rare exceptions, the aim of this field is largely to optimize indicators of well-being through AI-driven personalized insights, rather than treating/managing a diagnosed disease (15). A key exception is the continuous glucose monitor (CGM). The CGM has directly transformed diabetes management by enabling real-time, personalized feedback that directly links lifestyle behaviors to glucose outcomes, evolving the wearable into an active individualized intervention tool. CGM use has been shown to significantly improve glycemic control across diabetes types and is also used as behavioral intervention by providing immediate, individualized insights into how food, activity, and medication affect glucose levels (16, 17). However, the CGM benchmark deserves scrutiny: T1D populations are among the most adherent CGM users because they face acute, life-threatening consequences for lapses in glucose management, a level of extrinsic motivation that is largely absent in lifestyle-driven chronic disease prevention. Even within this highly motivated group, dropout and device fatigue are documented, suggesting that even gold-standard feedback loops require deliberate engagement design and cannot rely on clinical urgency alone (18, 19). An additional example includes Parkinson Disease, which reflects a practical application of P-DHTs as many of its movement-related symptoms are well captured from P-DHT-derived signals. For example, P-DHT measures have been able to capture symptoms such as gait disturbances, motor fluctuations, dyskinesia, and tremor demonstrating utility for both early detection (20) and management of the disease (21–23).

The P-DHT vision is to apply the CGM/Parkinsons model to other devices and areas of health—a tool that empowers the user to learn their personalized health-related modifiers and triggers and actionable suggestions to improve health. However, this becomes challenging without the direct, actionable biomarker feedback that CGMs provide or movement-related symptoms. The opportunity, which many P-DHT applications are attempting to do, is to track and follow multimodal (of different types) domains of health in the physiologic, psychologic, behavioural and environmental realms—encompassing ways to follow both health outcomes, but also, exposures, and other individual lifestyle factors that modify health. Through semi-continuous sensing, one can start to construct a personal portrait of granular, high resolution information, that over time, may give clues into key individualized factors that improve or worsen specific symptoms or disease states at the n-of-1.

Despite the rapid uptake of existing P-DHT tools with millions of users globally (24, 25), findings from systematic reviews demonstrate weak evidence that these tools can effectively impact hard clinical outcomes (outside the CGM—Diabetes case) (15, 26–28). Reflecting on the rise of the quantified self movement that touted personal wisdom through numbers—how far have we really gone since this theory began back in 2007 (29)? There was an early assumption that the passive data you can capture from P-DHTs could glean insight into an individual's health state ‘right now’. With some rare exceptions, this has proven difficult to achieve through passive data alone that tends to show weak, misleading and sometimes uninterpretable signals in isolation (30). Wearable/smart device data is inherently noisy from both signal accuracy and confounding. Heart rate variability (HRV) provides a hallmark example of this noisyness. Changes in HRV are nearly caused by everything—food and alcohol intake, smoking, noise, hormones, medications, temperature, activity and stress. As we are enabled to follow more granular measures of continuous health such as HRV, this comes with additional challenges relating to new ‘*micro-confounders’*—the many momentary external and internal factors that confound wearable signals and their association with health outcomes. There is now mounting evidence suggesting that understanding the context through which we experience day-to-day intrinsically and extrinsically, ‘the fabric of life’ is instrumental in isolating signal from noise in objectively measured P-DHT data (7, 31, 32), but the challenge isn't about collecting even more volumes of data—it's about collecting the right context data for an individual and this has not proven trivial. While ecological momentary assessments, and mobile app based, periodic patient reported outcome measures are often used to capture subjective ground truth, these alone are likely not sufficient to capture the momentary factors that drive P-DHT signal change.

### Where do we need to go?

To unlock P-DHTs’ preventive potential, we must evolve beyond tools that track, visualize, and nudge toward systems that actively contextualize signals within lived experience, moving from generic encouragement to individually meaningful, causally grounded insights that adapt as the person changes. What might help us get there includes: immersed and dedicated engagement strategies with new human-machine interaction paradigms (machine guided, not driven), micro-adaptive interventions including Just-In-Time (JIT) inference that offer short term results, and personalized data and insight summaries that are curated by the unique user. We also must consider how P-DHTs change the landscape in enabling individuals to participate in their own health and how to foster this change and reduce inadvertent consequences (33).

To capture the aforementioned context of micro-confounders requires deep and sustained engagement by the user so they can provide the needed labels to ground chaotic signals. Sustaining engagement over time remains one of the greatest hurdles for P-DHTs, with adherence typically declining after the initial novelty period despite promising short-term effects on behavior change (34, 35). The challenge lies in designing systems that transform self-tracking into self-understanding tools that dynamically adapt, recognize small successes in context from the user, and provide near-term, personally salient feedback that links micro-behavioural adjustments (e.g., shifting sleep timing) to tangible changes in how people feel. There is exceeding evidence on the power of extensive co-design with end users to promote better long-term engagement and efficacy success (36–41).

Micro-adaptive interventions offered in a continuous AI learning health loop, accompanied by Just-In-Time (JIT) prompts for inference incorporates a dynamic reflexivity that could further compound user engagement (14, 42–45). By continuously integrating P-DHT data and user-driven contextual signals, such systems infer an individual's momentary state and receptivity and then deliver brief, low-burden behavioral suggestions at the times when users are most likely to respond. Over time, the accumulation of many well-timed micro-behaviors, such as stress-regulation exercises, or sleep-supportive routines, could yield measurable changes in clinical and subclinical markers over time. This approach also encourages self-reflective insight about modifying factors that might be impacting day-to-day health that an individual is otherwise unaware.

Most on the market P-DHT tools offer some form of data visualization (i.e., symptom dashboard) that enables the user to observe certain data points over time; however, many lack customization and true personalization where the user can explore and make choices about when and how they see their data, and few center on identifying triggers, modifiers or symptoms. Further, many symptom dashboards are device specific—that is, you can only see data from one proprietary device, limiting the scope of complimentary multimodal data with a few exceptions again in the diabetes space that offer multi-device integration (46–48).

For P-DHTs to deliver on their healthcare prevention promise, the envisioned goal is to provide an individual with a personal portrait of multiple domains of health, layered by external and intrinsic factors that could modify an individual's personal health trajectory—a form of ‘personal compass’. The vision of this approach is that by interacting with such a personal ‘compass’, the user, over time, is empowered to gain insight into personal micro factors that improve or worsen day-to-day symptoms, providing a vehicle to show near-term cause and effect feedback. This shorter term reward, paired with the right UX/UI has the potential to better engage an individual in behavior change for the long-term. The potential final product of this ‘compass’ approach is a remote tool that empowers an individual to reflect on their health-related symptoms in relation to their unique lifestyle and environment with near-real time bio-feedback (Figure 1). A strength in this approach is in its universality. Such a tool could be leveraged both as a wellness and prevention tool—supporting individuals to navigate their individualized health particularly during periods of change that come with heightened risk such as the transition to emerging adulthood, and a number of health transitions such as perimenopause, but also a targeted intervention tool for numerous unique and multimorbid chronic conditions. While this approach is intrinsically centered on the individual, it not only attempts to facilitate a dialogue with oneself, but creates tools that could enhance the patient—healthcare provider dialogue. Further, by empowering a user to have a high resolution picture of their day-to-day health with mechanisms to share this information, this creates an opportunity to change the conversation about how we discuss, share and ask for support around ‘how we are feeling’.

**Figure 1:**
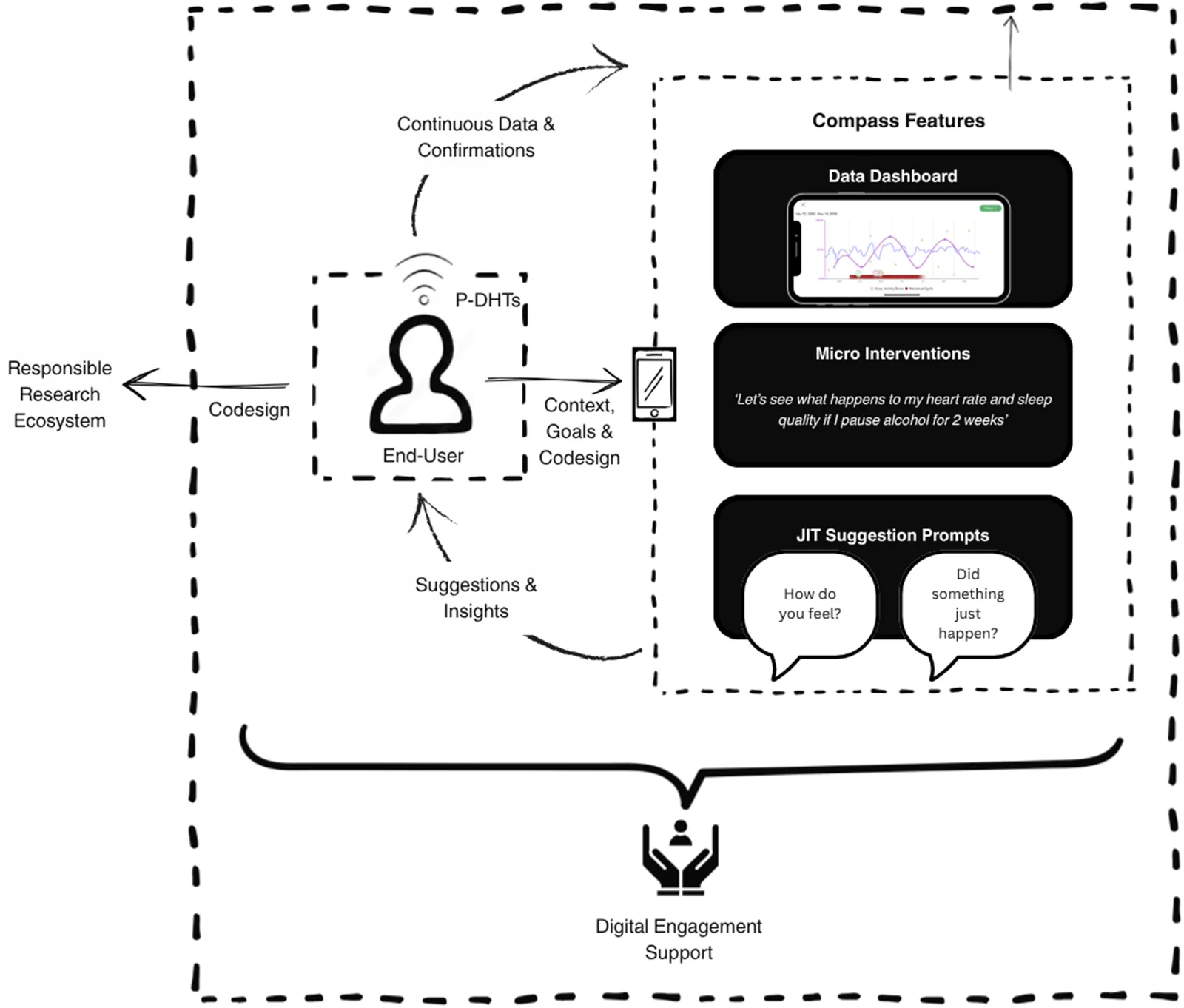
A personal P-DHT compass-HERE.

### Challenges

#### 1-vague biomarkers

As already observed in the case of CGMs for diabetic control, P-DHTs can work well when following symptoms and providing tailored lifestyle recommendations when there is a clear reactive, digital biomarker such as glucose. However, the extension of this model to most other symptoms and particularly elusive symptoms such as fatigue becomes more challenging (49). For example, there is no clear, validated digital biomarker for anxiety which is often related to crude behavioural proxies (e.g., daily movement, time spent at home) where accuracy is limited (50, 51). In the absence of a clear ground truth, this is where the strength of multimodal sensing comes into play, leveraging multiple P-DHT signals to verify an individual's presentation, augmented by the aforementioned context.

#### 2-equity and fairness

Scaling and widespread adoption of this approach raises important concerns about equity and fairness. Digital divides relating to the non-universality of access to wearables, smartphones, and stable internet disproportionately excluding individuals from lower socioeconomic backgrounds, rural areas, and older populations (52). Furthermore, algorithmic personalization depends on data that are often biased toward younger, wealthier, and healthier users, potentially amplifying existing health disparities (53). Privacy concerns and varying levels of digital literacy may further limit equitable participation. To ensure fairness, future designs must prioritize accessibility, transparency, and inclusivity in both data collection and user experience, ensuring that personalized digital tools empower, rather than stratify diverse populations. This will require deep codesign efforts with targeted end-user populations, and the right not-for-profit organizations backing these tools.

#### 3-analytic challenges

We now have technology to generate personalized visualizations, summaries, and explanations on demand. However, identifying which signals are meaningful, actionable, and likely to motivate behavior change is substantially harder. Most current AI methods are optimized to predict outcomes, whereas prevention-focused P-DHTs must identify factors that an individual can modify and that are likely to improve future health. This requires moving beyond prediction toward individualized causal understanding, determining not only what is associated with symptoms or outcomes, but what can be changed to meaningfully influence them. Developing robust methods for identifying personalized, modifiable drivers of health from noisy, multimodal longitudinal data remains a major unresolved challenge (54). Candidate methodological directions include N-of-1 causal inference frameworks (31), dynamic treatment regime estimation (55), and reinforcement learning approaches that treat each user as their own control (56, 57). None of these are mature enough for routine deployment at scale, but they define the analytic frontier that P-DHT evaluation must eventually engage (58).

A related challenge is that individuals are not static. The factors that drive engagement and behavior change vary across people and fluctuate within the same person over time in response to stress, mood, health status, and life circumstances. P-DHTs must therefore learn not only what information is important, but also how, when, and whether that information should be communicated. Importantly, systems cannot simply react to every momentary expression of frustration or disengagement. Information that has historically been beneficial for an individual may become temporarily unwelcome, while information that is well received may not ultimately lead to meaningful behavior change. Similar challenges have motivated the development of JIT Adaptive Interventions (JITAIs), which seek to tailor recommendations according to an individual's changing context and receptivity (58). Yet these decisions must often be made from sparse individualized data and under substantial uncertainty, requiring methods that can learn efficiently while adapting as both the individual and their circumstances evolve, an active area of AI research.

Determining whether these systems actually work presents an additional challenge. Much of the feedback available to P-DHTs is proximal, including engagement, satisfaction, or self-reported usefulness, whereas the outcomes that ultimately matter such as sustained behavior change, symptom improvement, and disease prevention may take months or years to manifest. Emerging evaluation frameworks such as microrandomized trials (MRTs), which repeatedly randomize intervention opportunities throughout daily life, provide a rigorous approach for estimating the short-term causal effects of adaptive interventions and are increasingly viewed as a foundation for evaluating personalized digital health systems (59, 60). However, translating improvements in proximal behavioral outcomes into continued health benefits is largely unresolved. A system that successfully increases engagement is not necessarily one that improves health.

#### 4-interoperability

Device companies operate in closed data ecosystems that involve proprietary silos that limit raw or minimally processed data access. This limits data interoperability across devices and the ability to harmonize data from multiple sources into a unified personal portrait. To overcome the interoperability challenges associated with closed P-DHT ecosystems, some collaboration from wearable companies will be needed to provide access to raw, minimally processed data so that harmonization and independent validation can be achieved across devices and populations. Alternatively, new open hardware/firmware approaches may be needed if this data access continues to be siloed.

#### 5-inadvertent consequences

There are fundamental unknowns about how this modern quantified self approach will work, where an individual is at the helm of their own health, potentially with a digital agent offering them AI driven advice—how will this feel? (61, 62). Despite the abundance of tools that enable self-tracking for health and wellness, there is a disproportionately small body of literature on how individuals actually perceive and interpret the information from self-tracking tools (63). Also of concern is the risk that an adaptive system risks optimizing for what is easiest to measure rather than what is most beneficial. For example, a system optimized primarily for engagement may learn to suppress clinically important but unwelcome information, reinforce avoidance behaviors, or prioritize short-term user satisfaction over long-term health goals. Similar concerns have been raised in the broader human-AI interaction literature, where optimizing systems around user feedback alone can lead to unintended and potentially harmful behaviors (64). Embedded safety rules should be a standard to avoid optimizing for a user response when a health concern is present. This could include safety alert messages based on both predefined criteria, but also when there is uncertainty as to what state the user is experiencing. Engagement data should be used as a tool for AI systems to decide how to communicate with a user, not whether clinically important information should be communicated. Developing methods that appropriately balance user preferences, clinical relevance, uncertainty, and long-term outcomes will be essential if P-DHTs are to evolve from passive monitoring tools into effective and trustworthy personalized health partners.

P-DHTs now produce unprecedented volumes of intimate physiological and behavioral data, raising significant concerns about nefarious use. Women face disproportionate risks, as data streams capturing menstrual cycles, fertility windows, sleep patterns, and location can be exploited for purposes far beyond their intended health applications (65). High-profile data breaches and the sale of reproductive health data to third-party brokers have demonstrated that app privacy policies frequently offer inadequate protection, leaving users vulnerable to surveillance, discrimination, and coercion. These risks are particularly acute in jurisdictions where reproductive rights are legally contested, where menstrual tracking data could be subpoenaed or weaponized to infer pregnancy loss or termination. The health data market around P-DHT data also presents concerns around use of patient P-DHT data. The commercialisation of healthcare infrastructures, where healthcare organizations partner with commercial entities for financial and/or infrastructure gain often takes financial advantage of patient data.

## Discussion

P-DHTs are already woven into daily life at scale, but scale without depth has not substantially moved the needle in improving hard health outcomes for individuals. The field now faces a clear inflection point: continue optimizing for engagement and surface-level wellness metrics, or make the harder methodological and design investments required to deliver genuinely personalized, causally grounded prevention.

Getting there requires progress on three fronts simultaneously. First, analytic: moving from associative models that predict outcomes to individualized frameworks that identify what a specific person can change to meaningfully alter their health trajectory drawing on emerging methods in N-of-1 causal inference, dynamic treatment regimes, and reinforcement learning. We need to construct new epistemic frameworks that explicitly acknowledge uncertainty, context, and the co-production of knowledge between human and machine. Second, evaluative: developing trial designs and proximal outcome measures that can rigorously link micro-behavioral changes to distal health benefits without waiting years for signals to emerge. Third, structural: ensuring that the tools built on these advances are co-designed with the end-users who stand to benefit most, are not gated by socioeconomic privilege, and are governed by data protections commensurate with the intimacy of what they collect.

The CGM has shown that when a wearable closes the loop between behavior and a biologically meaningful signal, sustained behavior change follows. The central challenge for the next generation of P-DHTs is to engineer that same closing of the loop for health domains where the signal is noisier, the motivation less acute, and the causal pathway less direct. If that challenge is met, the vision of a personal health compass, one that reflects not population averages but an individual's own modifiable drivers of health and moves from aspiration to achievable health prevention at scale.

## Data Availability

The original contributions presented in the study are included in the article/Supplementary Material, further inquiries can be directed to the corresponding author.
